# One Portal—with a Difference

**Published:** 1921-05-14

**Authors:** 


### One Portal—with a Difference.

It is related of Sir Isaac Newton, always much under the dominion of his domestic animals, that he was once discovered making a large hole in his study door for the cat to pass in and out, and a small one side by side for the kitten. It had not occurred to the philosopher that the kitten was not obliged to fill the entire aperture on her way through.

Something similar to this is the attitude of those doctors and matrons who have formulated objections to the breadth of the Draft Syllabus. They fear that in so wide and all-embracing a scheme the kitten—*i.e.*, the merely average nurse—will never get through.

Very grave apprehensions are filling the minds of the women responsible for training nurses in regard to the curriculum likely to be imposed in examinations for registration. Matrons of such widely different institutions as the Swansea General Hospital; the Liverpool Royal Infirmary; St. Bartholomew's Hospital; the Great Yarmouth Hospital; St. James's Infirmary, Balham; the Wolverhampton General Hospital; the General Hospital, Birmingham; the Royal Lancaster Infirmary are doubtful about the possibility of teaching all the subjects named in the Draft Syllabus to the rank and file, many of whom can only conform to an elementary standard of education.

The Draft Syllabus must be carefully differentiated from a curriculum supplied to the schools for examination purposes. But the trend of the discussions which have lately taken place makes it very clear that if registration is to be a success, and unite the entire profession, very careful heed must be taken of the limitations imposed by the natural abilities of probationers and by the teaching power in small schools. So far a syllabus of complete nursing education has been put forth, with indications of the manner in which it can be adapted to women of higher education, fit for teaching posts. But the imperative need of the moment is for a practical demonstration showing how the syllabus can be adapted to meet the needs of imperfectly educated women and ill-equipped schools. It may be taken as proved that many poorly educated women learn to be admirable nurses, trusted by doctors and beloved by their patients. The training-schools, large and small, cannot at the moment, and perhaps will never desire to, turn away women with a true vocation and every aptitude for nursing save the power of assimilating abstract terms. We must have it proved to us, as it was to Sir Isaac Newton, that the wide portal does not mean the exclusion of the inferior-sized animal. In this connection we direct our readers' attention to the *précis* from the General Nursing Council which we publish on p. 122. It does not go nearly far enough to allay the apprehensions which have been aroused.

